# Contextualizing context for synthetic biology – identifying causes of failure of synthetic biological systems

**DOI:** 10.1002/biot.201200085

**Published:** 2012-05-31

**Authors:** Stefano Cardinale, Adam Paul Arkin

**Affiliations:** Physical Biosciences Division, LBNL, Department of Bioengineering, University of CaliforniaBerkeley, CA, USA

**Keywords:** Complexity, Context, Environment, Gene expression, Synthetic biology

## Abstract

Despite the efforts that bioengineers have exerted in designing and constructing biological processes that function according to a predetermined set of rules, their operation remains fundamentally circumstantial. The contextual situation in which molecules and single-celled or multi-cellular organisms find themselves shapes the way they interact, respond to the environment and process external information. Since the birth of the field, synthetic biologists have had to grapple with contextual issues, particularly when the molecular and genetic devices inexplicably fail to function as designed when tested in vivo. In this review, we set out to identify and classify the sources of the unexpected divergences between design and actual function of synthetic systems and analyze possible methodologies aimed at controlling, if not preventing, unwanted contextual issues.

## 1 Introduction

Living systems are problem-solving systems. Even “simple” bacteria and viruses have solved the problem of surviving in every environment in which life can exist. They inhabit niches that are nearly completely isolated in the depths of the earth or in hyper-dense diverse, competitive communities in top soils or around plant roots. It is extraordinary how robust these organisms can be to changes in their environment [1]. No one appreciates this capability more than biological engineers who have yet to learn to design systems that are similarly flexible yet conserve designed function.

One of the common goals of synthetic biology is to make the design of new function vastly more efficient, safe, understandable, and predictable [2]. This field is likely to have a profound impact on chemical, pharmaceutical and material manufacturing, environmental and agricultural engineering, and health [3, 4]. A much cited barrier to predictability in design is context. Context, in this definition, is the environment in which a system finds itself. Organisms find themselves in environmental contexts where fluctuations of physical variables, such as temperature and osmolarity, and dynamical change in population density, diversity and interaction occur. These fluctuations impact physiology and fitness in complex ways. In fact, every process in the cell is also subject to context since each depends on the life of the host via direct and indirect interactions with cellular resources and components. They are also affected by the levels of both substrates and products, and the parameters of the cellular milieu, including the local redox potential, osmolarity, porosity/viscosity/crowding, and temperature.

We tend to assume that, for processes endogenous to the cell, most of these dependencies are evolutionarily optimized in some way to provide robust and effective cellular function and thereby fitness. It has also been conjectured that there might also be optimization for plasticity/evolvability [5]. Heterologous pathways have not had the advantage of long periods of co-evolution with other cellular substrates. They are generally subject to environments, such as bioreactors, that they have not experienced at length previously in the evolutionary history of the system. Thus, their function often suffers from uncontrolled/unpredicted interactions with the surrounding cellular context and environment. Unlike the mode in natural systems, the designer likely does not want the system to evolve, thus making plasticity undesirable. Further, the heterologous pathway itself is generally constructed from individual subsystems whose composition (physical and functional) is novel and therefore may generate spurious interactions unwanted or not predicted by the designer. We define all the implicit dependencies and spurious or unconsidered interactions mentioned above as context effects.

## 2 Subdividing context problems

Recently several authors have analyzed design strategies to directly integrate synthetic circuits with endogenous cellular processes [6], or to create systems that are more insensitive (i.e. robust) to variation in conditions [7]. Problems of context are barriers to the reliable function of biological pathways, and they are often intermixed with problems of robust and modular circuit design. In this review, we attempt to focus on the contextual mechanisms that are sources of variability/uncertainty that plague robust design. We also look at the contextual violations of interface definitions that challenge modular design of biological circuitry or circuit-host integration, and point to possible solutions for many of the effects. We define three broad classes of context: compositional context, host context, and environmental context.

Compositional context concerns the interface among biological components and the issues that arise in their physical and functional integration (Box 1a). Host context relates to the implicit dependence of a biological device on factors provided by the host organism for its operation. One consequence of this is a global coupling and competition among implanted and endogenous functions (Box 1b). Environmental context originates from variables that are asserted outside the host and that affect circuit function. Temperature, for example, is a variable that can directly affect heterologous circuit parameters as well as host functions, which, together with nutritive factors, stressors, and even other cells in the population, can affect the fitness of the host and modulate the effect of heterologous circuit function on that fitness (Box 1c).

**Box 1: Sources of context dependencies in synthetic systems**

**a. Compositional context**

- Physical integration of multiple regulators (e.g. transcriptional), genetic devices or polypeptides [8, 9, 12, 18].
- Approximate knowledge of RNA folding and cis-/trans-interaction of multiple regulators [13, 16].
- Functional composition of devices that leads to unexpected circuit failure [20–23].

**b. Host context**

- Parasitic interactions between transplanted and host-endogenous components [27, 28, 31, 33].
- Reliance on cellular components can affect host physiology [35–38], can depend on circuit “state” [40] and be host specific [43].
- Synthetic devices depend on cellular processes such as growth [46, 47], replication [53, 54] and partitioning at cell division [49, 50].

**c. Environmental context**

- Temperature and pH can directly affect cellular functions like transcription [57, 60, 61] or synthetic devices [62, 63].
- The host can mediate the effect of environmental context on the activity of synthetic components [70] or on population dynamics [72].

Below we review the evidence for the mechanisms underlying these different types of context effect, how they affect circuit and host function, and their implications for effective and reliable circuit design.

## 3 Compositional context

Compositional context is perhaps the easiest to address in improving the design of synthetic systems. Designers have a choice of what elements they use in their design and how they are physically instantiated and interconnected in a host (biological or otherwise). Thus, it may prove possible to make judicious choices in the elements we use to form the basis set for controlling ubiquitous cellular functions, although the biological principles of how to create such sets of parts are far from clear. Here we dissect examples of relevant mechanisms into physical and functional composition problems.

### 3.1 Physical composition

**Figure 1:**
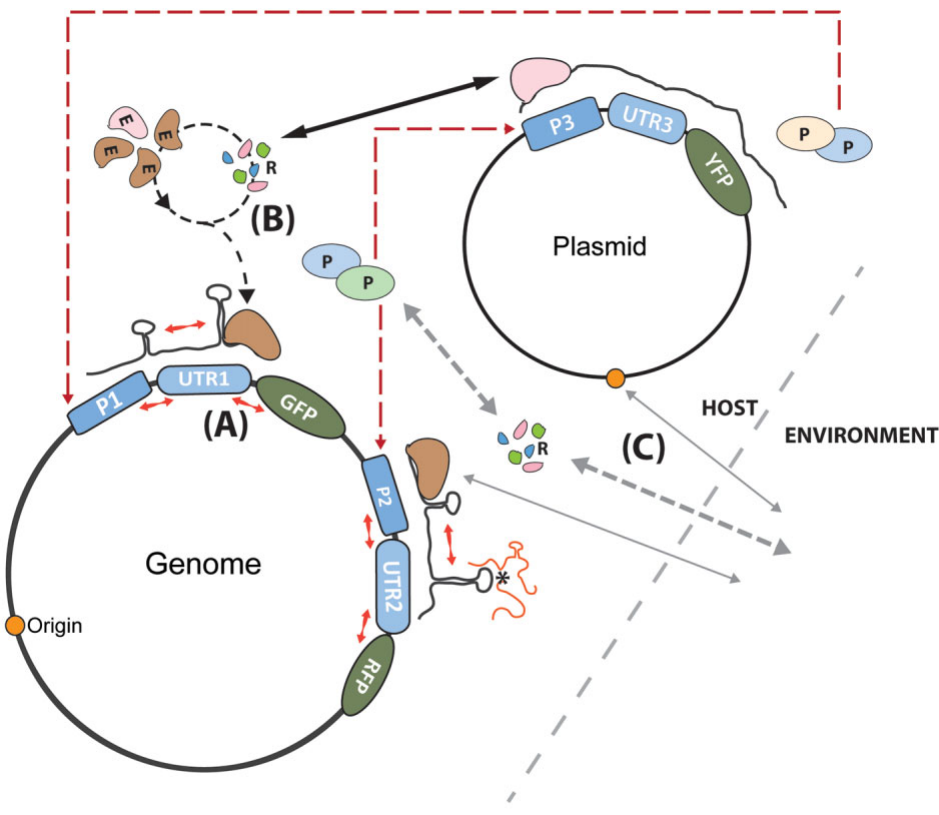
Sources of context effects and their connectivity. (**A**) Compositional context. 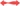, Physical composition: undesigned direct interaction between synthetic parts on the same molecule. 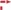, Functional composition: unexpected effects from module coupling, component titration or circuit “impedance”. (**B**) Host context. *, Parasitic interactions: undesirable interactions between synthetic and host endogenous components. 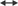, Host resources: synthetic devices often rely on host resources and machinery for functioning (R). Their production and partition can affect device activity. Alternatively, their depletion can affect host physiology, which can then affect the function of synthetic components. Also, host-specific variations of these endogenous components can be a source of context effects. 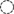, Host cell processes: synthetic devices are coupled to cellular fundamental processes such as replication, cell division and growth. (**C**) Environmental context. 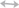, Direct effects: environmental factors such as temperature and pH can directly impact part activity. 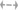, Host-mediated coupling: the environment defines the coupling circuit-host. For example, metabolic load can be sustainable or not in relation to the amount and quality of external nutrients.

In genetic circuits, the activity/effect of regulatory and expressed sequences arrayed on the same DNA molecule depends on their precise ordering, how they are linked across defined boundaries and their structural interaction. Similarly, domains on the same polypeptide or subunits within protein complexes often have activities that are mediated by defined interfaces and are affected by immediate sequence/structural context. When those interactions are “desirable”, they become design parameters. However, undesigned interactions can occur because of the often-necessary spatial co-localization of synthetic components, which can undermine their function. We define these unwanted, implicit interactions as physical context (Fig. 1A).

The meaning of *cis*-regulatory transcription factor (TF)-binding sequences in or near a promoter depends on the physical context through their spatial arrangement. In one study, Cox et al. [8] shuffled promoter elements to obtain a library of promoter architectures. The library generated a large range of expression strengths and logic gating from simple re-positioning of the operator sequences of a two-input promoter. In promoters that integrated binding of two repressors, the predominance of one or the other relied, often not intuitively, on their reciprocal position relative to the transcription start site [8]. If sufficiently elucidated, *cis*-regulation at the promoter will become a powerful design parameter for more complex regulation. However, as noted above, currently many spatial arrangements lead to surprising or unexpected results, making this a physical context problem. Promoters themselves can be composed together and tandem promoters have been used to produce gates with an OR logic. With just two inputs and one output, these OR gates could be assembled to construct all possible logic functions through compartmentalization of each circuit in a different strain of *Escherichia coli* [9]. In this case, while little interference was present between promoters for the majority of tandem combinations, the combination P_tet_ – P_BAD_ did not function as predicted. Why this combination failed was not obvious, although effects on DNA looping or occlusion of TF-binding sites were proposed [9]. In fact, if more complex transcriptional regulation becomes necessary, occlusion of regulator binding will probably gain importance as a design issue, and construction and screening of large part libraries will remain popular as an approach to design. Finally, multigene expression can still present a challenge because of poor termination at some Class I transcription terminators [10] or efficient transcription run-through in vivo [11]. These issues remain object of an intense engineering effort particularly to improve the efficiency of terminators for T7 RNA polymerase [12].

Integrating multiple regulatory inputs on RNA molecules can also be subject to undesigned interaction that could undermine functionality. Recently, Isaacs and co-workers exploited the versatility of RNA folding and the sequence specificity of RNA regulation to construct pairs of riboregulators that both repressed and activated translation from specific mRNA targets in vivo [13]. In this study, the observed parasitic interaction involved the 5' unstructured region of the engineered RNA riboregulator, which potentially formed alternative interactions that interfered with the riboregulator function [13]. In RNA-based devices, insulation of different functional elements can be the key for restoring part behavior. For example, modification of structural (stem) and spacer elements [14, 15], or self-cleaving ribozymes [16], can all be used to insulate RNA synthetic parts. In one study, Win and co-workers introduced a *cis*-acting aptazyme in the 3' untranslated region (UTR) region of the gene target of post-transcriptional regulation [15]. In this case, a designed sequence spacer was used to prevent potential interaction between the synthetic RNA device and other sequences on the target mRNA. However, while attempting to compose attenuators of different specificity, Lucks and co-workers reported that spacer sequences ranging from 100 to 1000 bp failed to provide sufficient insulation to tandem RNA attenuators, possibly because of strong structural interactions between RNA elements (upstream sense RNAs and downstream antisense RNAs). Instead, hammerhead ribozyme constructs were used to cleave off single attenuator elements and provided the necessary insulation to restore functionality [16].

Protein-protein interactions can be engineered to precisely direct signal transduction pathways by recruiting or sequestering regulators [17] or to artificially co-localize enzymes and substrates, e.g. to improve the yield of a biosynthesized product [18]. However, in this case, the co-localization of components (e.g. protein domains) can also lead to unwanted context effects. When heterologous protein-protein interaction domains were added to enzymes of the mevalonate pathway, the product titer increased stoichiometrically with the number of scaffold-recruiting, peptide-ligand domains. However, this effect reverted when the number of “scaffolded” domains increased above a certain level, possibly as consequence of enzyme misfolding or uncharacterized allosteric interaction caused by excess of ligand-peptide [18]. A precise definition of the number and relative stoichiometry of enzymes in designed scaffolds seems to be the key to avoid such compositional issues. However, the limits set by “microcompartment” formation, which is their most likely mechanism of function [19], might lead to further compositional problems.

### 3.2 Functional composition

Unlike physical composition, context effects in functional composition arise only when linking the output of one process to the input of another, and are not a direct consequence simply of physical interaction. Nearly all of these result from some competition for resources occurring between the output and the, possibly multiple, inputs. That is, the output of an upstream process is a molecule (or often more formally the production rate of this molecule) that is consumed as input by multiple downstream processes. To understand the effect for biochemical systems, a simple example would be a signaling molecule A that is produced at a constant rate and decays via a first order decay. Imagine two downstream processes that consume A to make B and C. The amount of A at steady state in this system is inversely proportional to the rates of conversion to these molecules. Thus, the steady-state rate of, say, B production depends on both these conversion rates. If conversion to C suddenly became faster, there would be less A at steady state and thus less B produced per unit time. Hence, the interconnection of A production to B production is affected by C production – the latter is in the “functional context” of B and vice versa [20]. This is not a desirable coupling in a modular design. Were all of these systems linear, there would be a relationship to the concept of low-input impedance that could lead to fan-out failure [21]. Recently, a formal mathematical definition of this type of context coupling in biochemical systems (called retroactivity), similar to the concept of impedance, has been developed [22, 23] and advances some approaches to mitigating these effects.

## 4 Host context

The task of synthetic biologists is constrained by the host organism that will contain their engineered designs. They depend explicitly or implicitly on the host resources and machinery for function, and ultimately their intermediate or final products are released in a cellular context with which they interact. It is the implicit interactions – those aspects of host function that are assumed to be constant and unlimited for the function of a design and to be insensitive to the operation of the circuit – that we define as host context (Fig. 1B). We classify undesigned host interactions into two classes: the first includes parasitic interaction in which unwanted molecular interactions between components of the heterologous circuit and host interfere with the function of both or either. The second class comprises functional interaction or coupling issues. The unaccounted dependence of circuit function on variable and limited host resources as well as cellular processes induces variable/unpredictable circuit behavior, coupling between components, or failure of circuit/host interface. Of course, the coupling is a closed loop. The functioning of the heterologous circuit can place a load on the host, affecting its fitness and consuming resources that would normally be available for endogenous processes. This, in turn, can create host resource limitations that restrict circuit function (e.g. by preventing optimal expression of an enzyme).

### 4.1 Parasitic interaction

Cellular pathways and circuits are defined, more or less, by a set of molecular species, the specific interactions among them, and the physical transformations permitted by the interactions (e.g. chemical reactions). However, it is also known that enzymes can be promiscuous [24], individual TFs can bind nonspecifically to DNA [25], and certain peptide-binding domains can bind many disordered target peptides with differing affinities [26].

Nonspecific binding and cross talk induce competition for the subject molecule among the specific modules that depend on them and the nonspecific consumers. TFs with new binding specificities have recently been obtained through directed evolution [27] or rational design [28, 29]. In at least one case the presence of undesigned parasitic interactions of the engineered regulators with the host was observed. Desai and co-workers [28] used an analysis of co-variation of TF and DNA target sequences to identify residues in the global regulator cAMP receptor protein (CRP) that confer different specificities, and constructed orthogonal regulator-operator pairs. Despite engineering several retargeted variants of CRP, they encountered unexpected cross-talk problems with the endogenous wild-type CRP, which was more promiscuous than the engineered regulators. In their work, Desai and co-workers suggested that the higher promiscuity of wild-type CRP towards operator sequences imported into *E. coli* from different species of bacteria originates from the lack of regulator-operator co-evolution in this organism [28]. Removing promiscuous binding or enzymatic activities by rational engineering, without compromising function, may be difficult given the long evolutionary selection for these attributes [30].

In an attempt to engineer the binding specificity of the lactose operon repressor LacI, Zhan and co-workers [29] used the extensive structural and functional knowledge of LacI protein-DNA interactions to design new repressor/operator pairs. These orthogonal regulators showed limited cross talk and could be assembled in various logic circuits. However, the authors also noted that the necessity for LacI repressors to oligomerize for repression, which relies on protein-protein interaction surfaces not involved in DNA binding, could be a source of undesigned cross talk and possible interference with host proteins of the same repressor family. In fact, in one study at least, strain-specific parasitic interactions with the host, e.g. host genome or proteome, have been confirmed for the LacI family of regulators. Wang and co-workers [31] constructed several logic circuits with orthogonal regulators imported from *Pseudomonas syringae.* This device did not function properly in five of seven different *E. coli* strains when engineered variants of *E. coli* promoters P*_lac_* and P*_bad_* were used as inputs, but it worked according to design when the exogenous promoter P*_lux_* was used in place of P*_lac_.* In this study, the authors uncovered an unexpected parasitic interaction of the *lacI* promoter with components of the LacI family of transcriptional regulators in *E. coli*, which undermined circuit functionality.

It is thought that protein complexes are subjected to both positive and negative selection during their evolution [32]. Positive selection could lead to increased binding affinity, but this occurs at the expense of possible synergistic, nonspecific binding to non-cognate partners. Alternatively, negative selection against binding to such unintended partners is thought to be an important driving force in the evolution of cognate versus non-cognate protein interactions, and could help to insulate signaling pathways in large paralagous protein families. In an elegant study, Zarrinpar and co-workers [33] demonstrated the effects of negative selection in the yeast *Saccharomyces cerevisiae.* The authors constructed chimeras of the yeast osmosensor protein Sho1 with its SH3 domain replaced with other yeast as well as distant metazoans SH3 domains. When the authors tested the amount of specificity stored in this interaction surface, they found that only the cognate domain recognized its peptide target sequence, and none of the paralogous domains could. However, SH3 domains from other organisms did bind the target peptide with varying affinities, possibly because they were not selected to not interact with the endogenous effector. The implication is that using these domains, heterologously, in *S. cerevisiae* would lead to undesired cross talk between the implanted system and the host. Such barriers to heterologous expression of genes have also been studied in a broader scope. Sorek et al. [34] attempted to pass 246, 045 genes from 79 prokaryotic genomes into *E. coli* and determined how many failed horizontal gene transfer. As expected, generally, proteins that are involved in fundamental cellular processes such as transcription and translation have little propensity to transfer. Intriguingly, beside several ribosomal proteins, a number of other classes of protein, including several membrane transporters and pumps, could not be transferred into *E. coli.* Possibly, these proteins could engage in spurious interactions with other cellular functions affecting cell viability [34].

### 4.2 Host loading: Reliance on host resources

Synthetic genetic circuits must function in a cellular environment, and normally use host resources and enzymes such as nucleotides, tRNAs or ribosomes for fulfilling their process requirements. Drawing from these pools of central resources can impact the normal homeostasis of the host cell. Expression of a heterologous protein can compete for limiting free ribosomes, which decreases the expression of other proteins [35, 36], and even reduces the number of functional ribosomes in the cell [36]. In particular, Dong and co-workers [36] showed that overexpression of β-galactosidase such that it comprised 30% of total cell protein completely halted cell growth. A more recent example shows that a particular coding sequence can interact with the host differentially and lead to indirect coupling between systems [37]. Here, the expression of two of three encoded proteins varied linearly with plasmid copy number. The third, GFP, fell off differently at high copy number apparently due to specific load from expression of this sequence. The load from *gfp* affected the linearity of the expression of the other sequences when placed on the same plasmid.

Models describing in detail the coupling between protein synthesis and cell homeostasis remain very important. The impact of protein overexpression on growth was recently found to be largely a consequence of re-allocation of ribosomes, constrained by a parameter describing nutritional capacity [38]. Interestingly, in their model Scott and co-workers [38] predict the presence in *E. coli* of a feedback mechanism that balances the amount of cellular ribosomes, possibly triggered by changes in the endogenous pool of tRNAs, in response to reduced translational activity. The constraints imposed by host resources are beginning to set bounds on the size and activity of synthetic circuits that may be expressed in different hosts and point to bottlenecks for which, in the future, we might design solutions. Importantly, the host organism also plays a strong role in determining which configurations of codons lead to optimal translation of the protein, particularly by defining the relative abundance of each charged tRNA [39].

It is likely that almost all heterologous circuits place some load on the cell, thereby affecting either its growth or its ability to carry out certain processes. The significance of the load on cellular resource and hence on fitness is, of course, both dependent on the “state” of the circuit and the “environment” of the cell. For example, in the yeast *S. cerevisiae*, Burrill and co-workers [40] engineered a positive-feedback device to sense the activation of DNA damage response and trigger a persistent output from the circuit that functioned as memory device. Nevertheless, circuits in the “ON” state could still affect the growth rate of activated versus non-activated cells, with the former being outgrown and diluted in the population, which could lead to partial loss of the long-term memory function of the device [40]. The design of this memory switch naturally leads to different host loading in the two states since the ON state expresses a protein at a high level and the OFF state does not. However, there are alternative switch designs in which load can be better balanced. DNA inversion-based memory switches in which state is held by the configuration of DNA after action of a recombinase could provide a lower load on the host [41, 42]. An invertase can flip a fragment of DNA to a different orientation, a mechanism that can serve directly as memory. Alternatively, if a protein must be expressed, the fragment might contain a promoter that expresses different proteins in each orientation, which assert similar loads on the cell and avoid subpopulations of cells with different growth characteristics.

As synthetic biologists expand the number of hosts in which they wish to transplant synthetic devices, being able to predict how each circuit will function in a new context or “environment” is clearly desirable. Nevertheless, genetic circuits can function differently even in different strains of the same species of bacteria. For example, in the work of Balagadde et al. [43], their population control circuit experienced unexpected oscillations over several days before reaching a lower steady-state population density. The cause of the oscillations was not clear and, of the two different *E. coli* strains tested, one – MC4100Z1 – had dampened oscillations, while the other – Top10 – had sustained oscillations for the whole length of the experiment. Furthermore, the two strains had a very different phenotypic response to the killer protein CcdB: MC4100Z1 showed a distinct stress phenotype, while Top10 did not [43]. These strain-specific dynamics are clear examples of unaccounted, contextual issues that arise from interfacing a circuit with the host genetic background. However, because MC4100Z1 and Top10 are so closely related, it is likely that the genetic basis of this difference in circuit response to the host can be mapped, and the mechanistic causes determined.

Predicting what cellular process, metabolite or resource will affect the function of a device is, for now, very difficult. In a surprising example of a load issue being beneficial to circuit function, Stricker et al. [44] designed a genetic oscillator that behaved more robustly and functioned at a wider range of inducer concentrations than predicted. They discovered that their circuit saturated the endogenous proteolytic machinery, inducing coupling among circuit components that was critical for robust oscillations to occur. There are likely a finite number of such key interaction points of heterologous circuit with cellular resources, and it is a key challenge to design against them or, alternatively, exploit them. It is more complicated, perhaps, to isolate a heterologous circuit from dependence on host “housekeeping” machinery and metabolism. One possibility that we have already mentioned is to physically confine different subsystems of a design to different cells [9]. With this method, both the metabolic load on the host and the interference of parasitic interactions can be limited by reducing the number of circuit components present in each cell type. Importantly, multicellular computing also has the benefit of reducing intercellular variability with a population-wide global output [9] and allowing division of labor and the execution of incompatible chemistries in the same circuit.

### 4.3 Growth-coupled context effects

Apart from host resources, synthetic devices in a cell are subject to the unavoidable recurrence of at least three cellular processes: growth, replication and partitioning. The dependence of the cellular macromolecular composition on growth rate has been a focus of intense investigation for over 30 years [45, 46]. In some bacteria, gene copy number is strongly correlated with growth; at higher growth rates multiple rounds of replication can simultaneously occur. Recently, a theoretical approach was adopted to analyze data of protein expression from different promoters in different media [47]. In this work, Klumpp and co-authors [47] established growth-rate dependencies of the transfer function of several types of gene circuits. Additionally, they extended the notion of “growth feedback” [48] identifying nonlinearity in host-circuit interaction that could lead to both expression and growth bistability in absence of positive feedback. In this case, the growth feedback enters in the form of the dilution of circuit intermediates and is dependent on whether the circuit is encoded on a plasmid or in the host genome. At the design stage, it is important to carefully consider whether gene circuitry is implemented on a multicopy plasmid or the host chromosome and which replication system is adopted because their copy number can be differently affected by growth feedback as recently demonstrated [47]. This is also necessary to counter issues associated with plasmid partitioning at cell division [49], and methods for controlling the dependence of circuit function on the abundance of encoding genetic material can be useful. In particular, Bleris and co-workers showed that certain types of circuit topology, such as the incoherent feed-forward loop (iFFL), adapt to changes in the DNA template abundance [50].

Plasmid maintenance can have unexpected, toxic consequences on the cell such as, for instance, increased glucose uptake and detrimental accumulation of metabolite intermediates [51]. As one further confirmation of the multiple, unexpected consequences of unbalanced metabolite pools, a dramatic change in charged tRNAs can interfere with plasmid replication control and lead to a dramatic increase in plasmid copy number [52]. Clearly, there is a positive feedback between plasmid-associated activities and host metabolism that can lead to unregulated “runaway” plasmid replication (uncontrolled plasmid amount) and have detrimental consequences on the protein synthesis apparatus or the integrity of the outer membrane [53, 54]. Accurate control of plasmid replication can go a long way toward limiting the metabolic burden of recombinant expression and plasmid maintenance on the host cell. Identifying and modeling feedback between indicators of growth and components of replication regulation will be important to implement copy-number stabilization in circuit design [55, 56].

## 5 Environmental context

Biological circuits and their hosts are subject to fluctuations in the environment. Resources may change, physical conditions such as volume and temperature may vary, and, of course, there may be other cells competing for resources in the environment. In addition, the cell is continuously sensing the environment; therefore, various problems for the function of a genetic circuit can originate from the very diverse interplay of host and environmental variables (Fig. 1C). We define environmental context as the unintentional coupling to variations in the environment that modifies the behavior of biological components either directly or indirectly through the mediation of the host organism.

### 5.1 Direct effects

Environmental factors can directly affect physical-chemical properties of biological components and, therefore, their function. Temperature can directly modulate transcriptional activity per se, without the involvement of *trans*-acting factors. For example, the promoter of bacteriophage λ P_L_ shows increased transcription when the temperature is lowered [57]. Temperature-dependent changes in the curvature of the DNA can favor the binding of activators [58] or repressors [59, 60]. Temperature effects need to be accounted for during design of gene circuits, particularly when RNA parts are used. Recently, efforts to characterize novel synthetic RNA “thermometers” were hindered by the observation that transcription/translation processes are somewhat more efficient at higher temperature, which can compromise proper characterization of other regulatory devices as well [61]. Riboswitches have been found to function under equilibrium conditions, in which temperature can be an important factor [62]. In a recent attempt to engineer riboswitches that functioned in bacteria other than *E. coli*, Topp and co-workers [63] determined that the temperature of the culture was an important variable to be considered in predicting riboswitch function in the bacterium *Magnetospirillum magneticum*, the optimal growth of which is at 30°C.

The pH of the growth medium is another important design variable in engineering biological parts. Probably the most investigated case is that of the quorum-sensing signal acyl homoserine lactone (AHL) molecule. Non-enzymatic, temperature- and pH-dependent hydrolysis of AHL is noticeable in aqueous solutions [64] (for a review see [65]). When the response to pH is predictable, this parameter can be used to effectively modulate circuit output. For example, the steady-state cell density reached by a bacterial population engineered with a population-control device could be tuned by changing the pH of the growth medium [66]. However, pH affected other parameters in the model such as cell growth and, unexpectedly, the toxicity of the killer protein CcdB [66].

### 5.2 Host-mediated environmental effect

Host resources and subsystem activity vary with the environment. The accessibility of common metabolite pools, the energy charge, and the availability of polymerases and ribosomes are all greatly affected by growth phase and available external nutrients [46, 67, 68]. Global regulators such as H-NS, IHF and Lrp in *E. coli* can change DNA accessibility in ways that impact circuit expression; proteases change their levels and thereby their ability to be saturated; and cells change volume, shape and other parameters that might affect circuit function. Likewise, the load imposed on a cell by a heterologous circuit is affected by the availability of external resources, and, in the extreme, can lead to changes in cell fitness that can have deep consequence for circuit function and stability. Undesigned interactions among different populations of cells, such as cross-feeding and cross-protection, can lead to unexpected dynamics that can ultimately alter circuit functionality.

One recent example of multi-level coupling with environmental regulation arose in the attempt to engineer a propionate-regulated promoter from *Salmonella enterica.* This promoter (P*_prpB_*) is activated by the regulator PrpR, which, in turn, is activated by 2-methylcitrate derived from the metabolism of propionate. Additionally, the promoter is regulated by the 3'–5'-cyclic adenosine monophosphate (cAMP)-CRP complex (e.g. catabolite repression) and possibly by the global regulator IHF, suggesting strong environmental sensitivity of propionate-dependent transcription regulation [69]. Indeed, P*_prpB_* was shown to be environment sensitive in a manner that depended on the particular strain and media used for expression. The promoter showed no activity in *E. coli* BL21 (BLR) cells and in Terrific broth (TB) medium but functioned properly when tested in Luria broth (LB) medium. In addition, when *E. coli* DH1 cells were used, the promoter was active in both LB and TB media [70]. The novel propionate promoter was not the only one to show strong host-mediated environmental effect. For example, the long-established *lac* promoter was repressed by the addition of 1% glucose to the medium, which is known to activate catabolite repression, and showed leaky expression in TB [70]. These data demonstrated that complex regulatory mechanisms used by bacteria to deal with nutrient availability, such as catabolite repression, could have strong and unexpected impacts on synthetic gene circuits when probed outside the experimental conditions used for optimization.

Microbes also modify their local environment by secreting metabolites and signals, originating complex subpopulation and subpopulation/interspecies dependencies through cross-feeding and cross-protection. Cross-feeding, trade-offs in growth-phase parameters, and spatial constrains within the culture, which affect the dilution of shared agents, are important factors involved in the emergence and maintenance of polymorphic subpopulations of bacteria (reviewed in [71]). Recently, during growth in a bioreactor, a polymorphic subpopulation of cells resistant to antibiotics such as norfloxacin or gentamicin was found to protect the majority of non-resistant cells by secreting the signaling molecule indole into the media, a molecule involved in stress tolerance in *E. coli* [72]. The design of synthetic devices with phases that can differently impact fitness trade-offs between cell subpopulations, such as a host-integrated memory/non-memory states [40] (often encoded on plasmids that can lead to the emergence of polymorphic, cooperative cells or “cheaters” [73]), will have to rely on careful analysis of complex population dynamics to engineer safer device containments and evolutionary and functional robustness during prolonged culture.

## 6 Concluding remarks

Synthetic devices are extraneous elements that are implanted in a cellular system that has used millions of years of evolutionary tuning to reach equilibrium. Currently, we cannot map genomes to account for all parasitic interactions that may occur between the vast array of synthetic biological parts being built and the organisms in which they will function. Neither we will probably, in the near future, be able to precisely model production and partitioning of all cellular resources in all different growth conditions for many types of organisms. However, as synthetic biologists expand the number of hosts in which they wish to transplant synthetic devices, especially to more complex biological systems found in higher eukaryotes, being able to predict how a circuit will function in a new context is clearly desirable. Context dependencies need to be part of the standard characterization of biological parts and some proposals to this end have already been made [74]. However, it remains an open challenge to create the physical map of where and how such dependencies arise with similar precision to that found in electrical or mechanical engineering. Results from genome-scale studies on gene-phenotype interaction suggest that, although host and immediate context effects might be complex, they are finite [75, 76], and could likely be dealt with by formal design. In fact, as the studies cited above have demonstrated, diagnosing the failures of designed systems to meet specification is leading to deeper understanding of different types of molecular mechanisms, the nature and limitations of cellular resources, and the indirect coupling among cellular subsystems, and has hinted at undiscovered processes.

Recently, it has been pointed out that it may be possible to design genetic circuit “probes” to precisely dissect these new cellular processes [6]. Further, new theories of robust design and design frameworks for biological insulation elements are emerging in the toolkits of designers [7]. The synthetic biology community does not lack engineered biological parts or experimental tools to identify and characterize these contextual effects; hence, the field is ready to systematically address these issues and improve predictability in the design of biological systems.

Dr. **Stefano Cardinale** is a postdoctoral researcher at Lawrence Berkeley National Laboratory and University of California, Berkeley (Berkeley, USA). During his PhD program at the University of Edinburgh (Scotland, UK), Wellcome Trust Center for Cell Biology, he worked on the construction, manipulation and application of Synthetic Human Chromosomes in human tissue cells. This work led to two patent applications and several scientific publications. In his carrier, Dr. Cardinale has received an EMBO Short-Term Fellowship to pursue research on nuclear structures in human cells and a Darwin Trust Scholarship for his graduate studies. His current research focuses on the effect of each gene of the E. coli genome on cellular physiology and the activity of synthetic genetic circuits to identify key sources of failure of synthetic devices in bacteria and to improve predictable design of synthetic systems. His research interests are Systems and Synthetic Biology, and biotechnology.

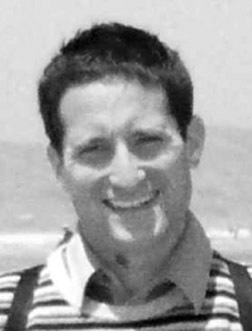

Prof. **Adam Arkin**, Director of the Berkeley Synthetic Biology Institute and of the Physical Biosciences Division at Lawrence Berkeley National Laboratory (LBNL), is the Dean A. Richard Newton Memorial Professor in UC Berkeley's Department of Bioengineering. He is also the Chief Executive and Science Officer of the Department of Energy Systems Biology Knowledgebase, technical co-manager of the large scale Ecosystems and Networks Integrated with Genes and Molecular Assemblies (ENIGMA) program, Director of Bioinformatics at the Joint Bioenergy Institute, and Co-director of the BIOFAB (International Open Facility Advancing Biotechnology). His research centers on uncovering the evolutionary design principles of cellular networks and populations, and exploiting them for applications. He and colleagues are developing a framework to facilitate applications in the fields of health, environment, and bioenergy by combining comparative functional genomics and genetics, quantitative measurement of cellular dynamics, systems biological modeling of cellular networks, and cellular design. He has been a member of the UC Berkeley faculty since 1999, and earned his PhD in physical chemistry from Massachusetts Institute of Technology (MIT). He was named a Fellow of the American Academy of Microbiology in 2007, and has been profiled in Time Magazine as a “future innovator”. He was a member of the founding class of the MIT Technology Review Young Innovators program. He is the author or co-author of over 190 scientific papers and reviews.

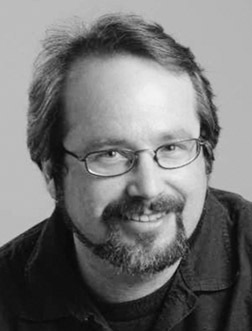

## Abbreviations

TF: transcription factor
CRP: cAMP receptor protein
TB: terrific broth
